# Association Between Frailty and Development of Alzheimer's Disease–Related Dementias

**DOI:** 10.1093/geroni/igaa057.137

**Published:** 2020-12-16

**Authors:** Chih-Ying Cynthia Li, Brian Downer, Lin-Na Chou, Kenneth Ottenbacher, Kyriakos S Markides, Soham Al Snih

**Affiliations:** The University of Texas Medical Branch at Galveston, Galveston, Texas, United States

## Abstract

Frailty is associated with an increased risk for Alzheimer’s disease and related dementias (ADRD). However, this association has not been investigated in older Mexican Americans; a population that is high-risk for frailty and ADRD. This study investigated the association between frailty and the development of ADRD over 9-year period. We analyzed 860 Mexican Americans ≥76 years old of the Hispanic Established Populations for the Epidemiological Study of the Elderly (Hispanic-EPESE) who have been linked with Medicare claims data. Survey data from Wave 6 (2007/08) was used to categorize participants as frail (either pre-frail or frail) or non-frail according to the Fried phenotype. The main outcome was ADRD diagnosis after Wave 6 interview. ADRD status was determined using the Chronic Conditions Segment of the Master Beneficiary Summary File. We estimated ADRD disease-free probability during 2007-2016 using midpoint of interval data method stratified by frailty status. Mean age of the study sample was 83.2 years (SD=4.4) and 59.3% were female. We found individuals who were frail had less ADRD-free months (46.5; SD= 36.5-52) compared to those who were non-frail (66.0; SD= 47.5-120). Individuals with a score of less than 21 points on the Mini Mental Status Exam had greater risks of ADRD development (Odds Ratio=1.35, 95% CI= 1.05-1.74) compared to their counterpart, after controlling mortality as a competing risk. Our results suggest being pre-frail, frail or cognitively impaired are risk factors for ADRD in community-dwelling older Mexican Americans.

